# Beta‐cell excitability and excitability‐driven diabetes in adult Zebrafish islets

**DOI:** 10.14814/phy2.14101

**Published:** 2019-06-03

**Authors:** Christopher H. Emfinger, Réka Lőrincz, Yixi Wang, Nathaniel W. York, Soma S. Singareddy, Jennifer M. Ikle, Robert C. Tryon, Conor McClenaghan, Zeenat A. Shyr, Yan Huang, Christopher A. Reissaus, Dirk Meyer, David W. Piston, Krzysztof Hyrc, Maria S. Remedi, Colin G. Nichols

**Affiliations:** ^1^ Department of Cell Biology and Physiology Washington University in St. Louis St. Louis Missouri; ^2^ Department of Medicine Division of Endocrinology, Metabolism, and Lipid Research Washington University in St. Louis School of Medicine St. Louis Missouri; ^3^ Center for the Investigation of Membrane Excitability Diseases Washington University in St. Louis School of Medicine St. Louis Missouri; ^4^ Institute of Molecular Biology/CMBI Leopold‐Franzens‐University Innsbruck Innsbruck Austria; ^5^ Department of Pediatrics Washington University in St. Louis School of Medicine St. Louis Missouri; ^6^ Present address: Department of Cardiology Renmin Hospital of Wuhan University Wuhan China

**Keywords:** Calcium channels, insulin secretion, K_ATP_, metabolism, pancreas, zebrafish

## Abstract

Islet *β*‐cell membrane excitability is a well‐established regulator of mammalian insulin secretion, and defects in *β*‐cell excitability are linked to multiple forms of diabetes. Evolutionary conservation of islet excitability in lower organisms is largely unexplored. Here we show that adult zebrafish islet calcium levels rise in response to elevated extracellular [glucose], with similar concentration–response relationship to mammalian *β*‐cells. However, zebrafish islet calcium transients are nor well coupled, with a shallower glucose‐dependence of cytoplasmic calcium concentration. We have also generated transgenic zebrafish that conditionally express gain‐of‐function mutations in ATP‐sensitive K^+^ channels (K_ATP_‐GOF) in *β*‐cells. Following induction, these fish become profoundly diabetic, paralleling features of mammalian diabetes resulting from equivalent mutations. K_ATP_‐GOF fish become severely hyperglycemic, with slowed growth, and their islets lose glucose‐induced calcium responses. These results indicate that, although lacking tight cell‐cell coupling of intracellular Ca^2+^, adult zebrafish islets recapitulate similar excitability‐driven *β*‐cell glucose responsiveness to those in mammals, and exhibit profound susceptibility to diabetes as a result of inexcitability. While illustrating evolutionary conservation of islet excitability in lower vertebrates, these results also provide important validation of zebrafish as a suitable animal model in which to identify modulators of islet excitability and diabetes.

## Introduction

Electrical activity is the essential trigger of insulin secretion in mammalian *β*‐cells (Koster et al. 2006). At low plasma [glucose], ATP‐sensitive potassium (K_ATP_) channels are open, hyperpolarizing the cell membrane and keeping voltage‐dependent calcium channels (VDCCs) closed, thereby inhibiting secretion. A rise in plasma glucose results in enhanced *β*‐cell glycolysis and oxidative phosphorylation, and increases the [ATP]/[ADP] ratio. This in turn results in closure of K_ATP_ channels and, because of electrical coupling via gap junctions (Benninger et al. 2008), uniform depolarization of the *β*‐cell syncytium and calcium influx through VDCCs, which triggers pulsatile insulin release. The essential role of this coupling is illustrated by the fact that gain‐of function mutations in K_ATP_ channels cause diabetes, whereas loss of channel function reciprocally causes hyperinsulinism and hypoglycemia (Remedi and Koster 2010).

K_ATP_ channel genes are not present outside the vertebrates, and the evolutionary extent and origins of excitability‐dependent insulin secretion, although well‐elucidated in mammals, has not been well‐studied even in lower vertebrates. This is important, both from an evolutionary perspective, and to provide novel insights to the process. We have recently shown that K_ATP_ channels are expressed in *β*‐cells within the zebrafish (*Danio rerio*) islet, that they are functionally similar to their mammalian orthologs, and that activation of these channels by the drug diazoxide can similarly alter glucose tolerance (Emfinger et al. 2017). Previous studies have suggested a role for K_ATP_ channels in early islet responses to overnutrition: activation of K_ATP_, either pharmacologically or with inducible transgenes, increases *β*‐cell growth in response to excess nutrients (Li et al. 2014). However, direct assessment of zebrafish islet function is lacking, and whether alterations in excitability can drive persistent changes in glucose control in zebrafish, remains unknown. We recently showed that intracellular [Ca^2+^] is glucose‐sensitive in embryonic zebrafish islets (Lorincz et al. 2018). Here we characterize the glucose‐sensitivity of intracellular [Ca^2+^] in adult zebrafish islets, and show that it is similar to mammals, although unlike in mammalian islets (Bavamian et al. 2007), *β*‐cells appear to function as independent units, such that Ca^2+^ transients are not well‐coupled between *β*‐cells. We further show that transgenic expression of K_ATP_‐GOF mutations blocks glucose‐dependent [Ca^2+^] elevations, resulting in severe hyperglycemia, paralleling the consequences of *β*‐cell inexcitability in mammals.

## Methods

### Ethical approval

All animal procedures were approved by the Washington University in St. Louis Instiutional Animal Care and Use Committee.

### Generation of transgenic fish

Cytosolic gCAMP6s expression in zebrafish islet *β*‐cells was achieved using constructs optimized for tol2‐transposase insertion (Kwan et al. 2007). The construct was generated using gateway recombination of plasmids containing the promoter, gCAMP6s cDNA, and poly‐A stop sequence (sequence for the Tg(‐1.0ins:gCAMP6s)^stl441^ transgenic fish (cgCAMP6s fish). Constructs for generating fish which conditionally express gain‐of‐function K_ATP_ channels only in islets (Tg(‐1.0ins:LoxP_mCherry_polyA_LoxP,Kir6.2(K185Q,ΔN30)‐GFP)^stl443^, Fig. 5A) were created as per the insulin‐gCAMP6s vector. The mutant Kir6.2 subunit gene was cloned from an existing vector containing a gain‐of‐function mutation in the subunit (Kir6.2(K185Q,ΔN30)‐GFP) (Koster et al. 2000).

For each of the constructs above, transgenic fish were created as follows: 2 nL of injection solution containing 25 ng/*μ*L of construct and 25 ng/*μ*L of Tol2 transposase RNA were injected into AB zebrafish embryos at the single‐cell stage. The pDestTol2CG2 vector contains eGFP expressed under the cardiac myosin light‐chain promoter as a transgenesis marker, permitting detection of subsequent founders by visible green fluorescence in the heart. For heat shock induction in larvae, larvae were placed in 20 mL glass scintillation vials at 40–70 larvae/vial and heated at 37°C from day 1–5 pf in a water bath for 3 h/day. For adult induction, fish were transferred to glass beakers (7 fish/500 mL) with air stones and placed in a 37°C water bath for 3 hr/day for 2–10 days.

### Animal lines and maintenance

In addition to the transgenic lines above, we used AB wild‐type fish as well as previously described *β*‐cell‐specific eGFP‐expressing fish (Tg(−1.0ins:eGFP)^sc1^) (Moss et al. 2009), membrane‐tethered insulin promoter‐driven gCAMP6s fish (Tg(ins:lynGCaMP6s,ins:H2B:RFP) (Kimmel and Meyer 2016), and ubiquitin‐gCAMP6s fish (Chen et al. 2017). All fish lines were housed in the Washington University Zebrafish Facility under standard conditions, the details of which can be found at: http://zebrafishfacility.wustl.edu/documents.html.

### Gene expression analyses

RNA and cDNA were prepared using Qiagen RNeasy mini kit and ThermoFisher High‐Capacity cDNA reverse transcription kit respectively. Previously described protocols were used to isolate islet (Emfinger et al. 2017) and brain (Lopez‐Ramirez et al. 2016) tissues. Heart tissue was collected with forceps. PCR was performed with previously published primers for connexin 35b (Carlisle and Ribera 2014).

### Western blot protein detection

Zebrafish islets and brain were homogenized in cell lysis buffer (CST, MA) containing complete protease inhibitors (Roche Applied Science, Mannheim, Germany). 25 *μ*g of total protein lysate was loaded per lane. Blots were incubated overnight with the following antibodies: GAPDH (1:1000, CST, MA), Connexin 35 (1:250, #MAB3045, EMD Millipore, MO). Blots were washed and probed with RDye infrared fluorescent dye‐labeled secondary antibody conjugates (1:10,000; LI‐COR biotechnology). Fluorescence intensity was quantified by image studio Lite (LI‐COR biotechnology).

### Electrophysiological analyses

Islets were harvested and dispersed into individual *β*‐cells as described (Emfinger et al. 2017). Ca^2+^ currents were recorded in whole‐cell patch‐clamp mode. The extracellular solution contained (in mmol/L): NaCl 137, CsCl 5.4, CaCl_2_ 1.8, MgCl_2_ 0.5, Glucose 10, HEPES 5, NaHCO_3_ 3, NaH_2_PO_4_ 0.16 (pH 7.4). The patch pipette was filled with a solution containing (in mmol/L) CsCl 130, TEA‐Cl 20, MgCl_2_ 1, CaCl_2_ 0.5, K_2_ATP 3, EGTA 5, HEPES 10 (pH 7.4). The resistance of the pipettes was 2–3 MΩ. K_ATP_ currents were also recorded in whole‐cell patch‐clamp mode. The extracellular solution contained (in mmol/L): NaCl 137, CsCl 5.4, CaCl_2_ 1.8, MgCl_2_ 0.5, Glucose 10, HEPES 5, NaHCO_3_ 3, NaH_2_PO_4_ 0.16 (pH 7.4). The patch pipette was filled with a solution containing (in mmol/L) CsCl 130, TEA‐Cl 20, MgCl_2_ 1, CaCl_2_ 0.5, K_2_ATP 3, EGTA 5, HEPES 10 (pH 7.4). Whole‐cell voltage clamp recordings of K currents and excised inside‐out patch‐clamp recording of K_ATP_ currents was also carried out as previously described (Emfinger et al. 2017). In all experiments, pipette resistance was typically 2–3 MΩ, data were filtered at 1 kHz and recorded at 3 kHz. Current records were analysed using pClamp.

### Ex‐vivo microscopy of adult zebrafish islet calcium

Islets were isolated as described (Emfinger et al. 2017). Glass‐bottomed 35 mmol/L dishes (MatTeK) were coated with 1% agarose, and glass pipette tips were used to remove a section of agarose at the plate center, creating a well. Individual islets were transferred to wells and immersed in pH 7.4 Krebs Ringer's solution buffered with HEPES (KRBH) containing 2 mmol/L glucose. The KRBH base solution consisted of (in mmol/L): NaCl 114, KCl 4.7, MgSO_4_ 1.16, KH_2_PO_4_ 1.2, CaCl_2_ 2.5, NaHCO_3_ 5, and HEPES 20, with 0.1% BSA. Solutions of varying glucose concentrations were flowed into the plate chamber through lines running into and out of the chamber (Fig. 2A). Bulk islet data were captured using a Zeiss Axiovert 200M microscope equipped with a Lambda DG‐4 illumination system and EM‐CCD camera and a Till photonics microscope with PolyChrome V monochromator and cooled CCD camera in the CIMED Live Cell Imaging Core (https://research.wustl.edu/core-facilities/cmed-live-cell-imaging-core/). Time lapse images used 100 msec exposure at an interval of 500 msec. For single‐cell comparisons, high resolution images were captured using a Nikon Spinning Disk confocal microscope (a motorized Nikon Ti‐E scope equipped with PerfectFocus, a Yokagawa CSU‐X1 variable speed Nipkow spinning disk scan head, and Andor Zyla 4.2 Megapixel sCMOS camera) at the Washington University Center for Cellular Imaging (http://wucci.wustl.edu/). Images of ubiquitin‐gCAMP6s fish islets were collected on a Zeiss LSM 880 Airyscan confocal microscope equipped with two non‐descanned detectors for two‐photon imaging, also at the Washington University Center for Cellular Imaging. Time‐lapse images used 100 msec exposure at 1 sec intervals. All images were analyzed in Fiji (Schindelin et al. 2012). To correct for movement in x‐ and y‐planes, images were stack registered (using StackReg, rigid body) in Fiji before analysis. All calcium image data are presented as change in fluorescence intensity relative to baseline fluorescence intensity. Because the maximum excitability of an islet or *β*‐cell can vary, and the intensity of islet fluorescence can vary, glucose responses are shown normalized to the change in fluorescence in response to KCl (showing maximum excitability due to islet depolarization). For determining trace cross‐correlation and synchronicity, ROI measurements were analyzed using PeakCaller in MATLAB (Artimovich et al. 2017). The KCl response was excluded from segments in which cross‐correlation analysis was performed, to capture the responses to glucose only.

### Chemicals

All salts, amino acids, and other compounds were purchased from Sigma, except where indicated above.

### Statistics

Statistical analyses were made in GraphPad prism. Except as noted, each data set was tested for deviation from normal distribution (D'Agostino‐Pearson). For multiple group column data comparisons, data were analyzed by ANOVA, followed by Tukey's post‐tests where normality assumptions were met. In comparisons of two groups, Student's *T* test with Welch's correction was used. In cases of non‐normal distributions, the Kruskal–Wallis (more than two groups) or Mann–Whitney (two groups) tests were used. All values are indicated as mean ± SEM, except where noted. For dose–response curves, data were fitted using nonlinear regression.

## Results

### Zebrafish islets express l‐type calcium channels

**Figure 1:**
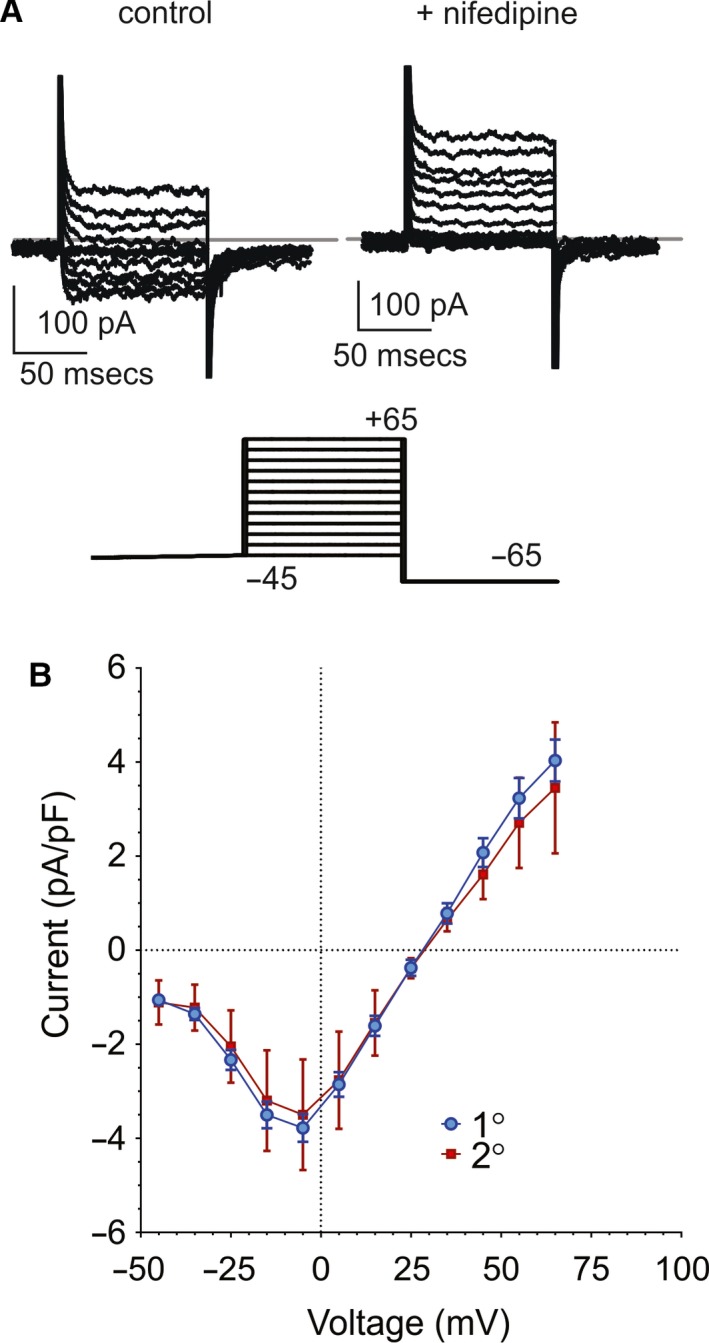
l‐type Ca currents in zebrafish *β*‐cells. (A) Representative recordings of whole‐cell Ca^2+^ currents (top) in response to voltage steps (bottom) from −45 mV holding potential to −45 to + 65mV are inhibited by (10 *μ*mol/L) nifedipine (right). Gray line in upper panels indicates 0 pA. (B) Summary of current–voltage relationship for calcium currents in isolated zebrafish *β*‐cells (14 total primary (1°) and 2 secondary (2°) islet cells).

The fish genome contains orthologs of the calcium channels found in mammalian islets, and RNA encoding these channels is present in islets (Sidi et al. 2004; Zhou et al. 2008; Sanhueza et al. 2009; Tarifeño‐Saldivia et al. 2017), but functional demonstration of Ca^2+^ channels in fish islet cells is lacking. Whole‐cell voltage‐clamp recordings from isolated zebrafish *β*‐cells reveal nifedipine‐sensitive l‐type calcium currents (Kuryshev et al. 2014; Striessnig et al. 2015) (Fig. 1A), with current/voltage profiles (Fig. 1B) very similar to those of mammalian l‐type calcium currents (Lipscombe 2002; Mangoni et al. 2006), the observed major VDCCs in mammalian islets. The zebrafish pancreas develops from the endodermal germ layer comprising endocrine and exocrine tissue and is conserved from mammals to fish. An early ‘primary’ islet forms within the first day of development (Argenton et al. 1999; Biemar et al. 2001), and additional smaller duct‐related “secondary” islets form as development progresses (Chen et al. 2007; Parsons et al. 2009). Similar currents were identified in both primary islet and secondary islet cells (Fig. 1B).

### Adult Zebrafish islet calcium is glucose responsive

**Figure 2:**
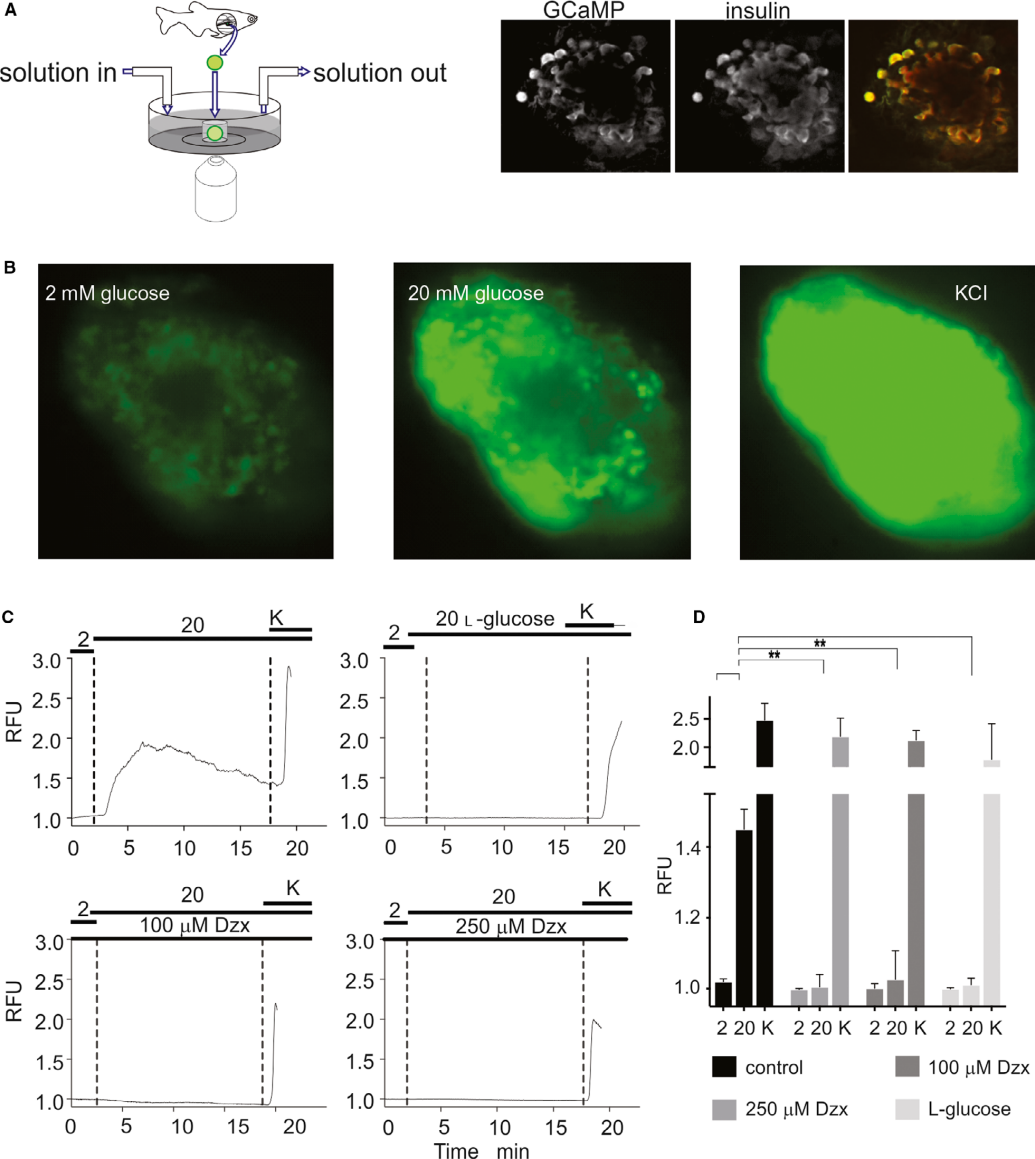
Intracellular [Ca^2+^] in adult islets is glucose‐sensitive. (A, left) Islets were imaged in microchambers (~4 *μ*L) cored out of agar, on the bottom of a petri dish. Flow of bulk solution into and out of the dish (~1 mL) was controlled as indicated. (right) GCaMP fluorescence and anti‐insulin staining in representative islet, together with overlay (GCamp fluorescence green, anti‐insulin red). (B) Individual frames of islets at low (2 mmol/L) and high (20 mmol/L) glucose (middle), and in 20 mmol/L glucose plus 30 mmol/L KCl (right). (C) Representative fluorescence traces from individual islets, normalized to initial fluorescence (relative fluorescence units, RFU), during transitions from low glucose to 20 mmol/L d‐ or l‐glucose, and 20 mmol/L glucose plus 30 mmol/L KCl, in absence or presence of diazoxide, as indicated. (D) Summary of calcium responses to high 2 or 20 mmol/L d‐ or l‐glucose, or 20 glucose plus 30 mmol/L KCl, in absence or presence of diazoxide, as indicated from experiments as in C (*N* = 6–10 in each case). (**) *P* < 0.05.

**Figure 3:**
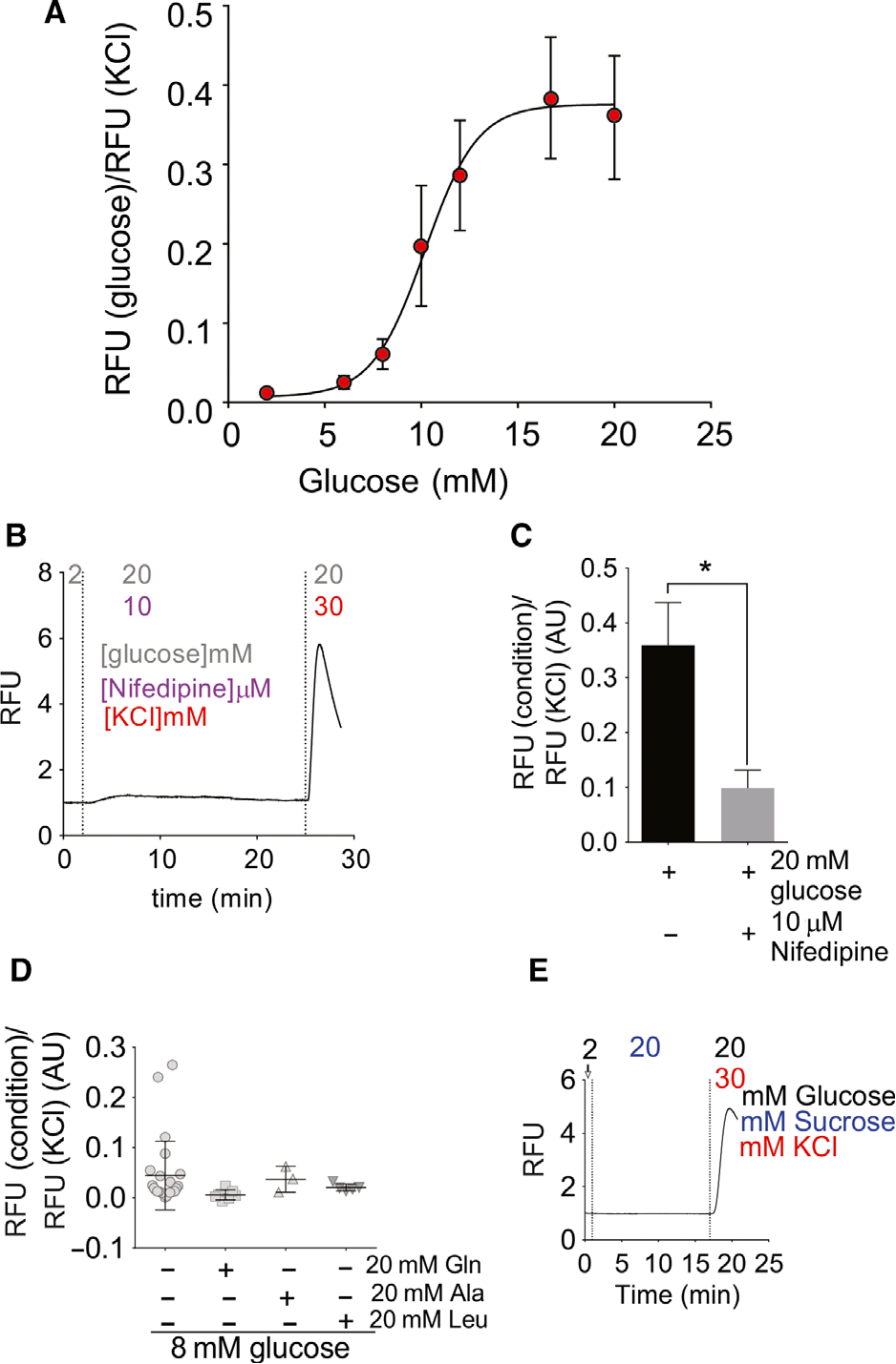
Glucose‐ and amino acid‐sensitivity of intracellular [Ca^2+^]. (A) Summary of [glucose]‐GCamp fluorescence response relationship from experiments as in Figure 1, with peak fluorescence at the indicated glucose concentrations normalized to the maximum fluorescence elicited by KCl depolarization (*N* = 8–17 islets/concentration). (B) Representative trace showing islet treated with 20 mmol/L glucose in presence of 10* μ*mol/L nifedipine. (C) Summary of calcium responses to high (20 mmol/L) glucose in absence (*N* = 8) or presence of nifedipine (*N* = 10). (*) *P* < 0.05. (D) Calcium responses to 8 mmol/L glucose in absence or presence of additional amino acids (normalized to maximum fluorescence in KCl. (E) Representative trace for islet calcium responses to sucrose (20 mmol/L).

To probe intracellular calcium levels and potential responsivity to glucose in zebrafish *β*‐cells, we generated Tg (‐1.0ins:gCAMP6s)^stl441^ fish expressing cytosolic gCAMP6s driven by the zebrafish insulin promoter. Ex vivo isolated islets from these cGCAMP6s fish show co‐localization of cGCAMP6s fluorescence and insulin staining (Fig. 2A). Figure 2B shows representative snapshot images and timecourses of cGCAMP6s fluorescence in the presence of low glucose and after switch to high (20 mmol/L) glucose, and then after addition of KCl. The response is specific to metabolizable d‐glucose, since there was no response to 20 mmol/L l‐glucose (Fig. 2C and D), and is sensitive to diazoxide (Fig. 2C and D), indicating that it involves closure of K_ATP_ channels (see below). The sigmoidal [glucose]‐dependence (Fig. 3A), obtained from similar experiments with switches to intermediate [glucose], is similar to that seen in mammalian islets, with EC_50_ of ~10 mmol/L glucose, slightly higher than the typically reported 5–9 mmol/L for mouse and rat (Antunes et al. 2000), or human (Henquin et al. 2006) islets. Consistent with the nifedipine sensitivity of calcium currents, the glucose‐induced calcium responses are abolished by the addition of 10 *μ*mol/L nifedipine (Fig. 3B and C). In contrast to mammalian islets (Henquin et al. 2006; Liu et al. 2008) and embryonic zebrafish islets (Lorincz et al. 2018), the amino acids glutamine, alanine, and leucine caused no activation in the presence of threshold (8 mmol/L) glucose (Fig. 3D). Finally, islets did not show any response to sucrose (Fig. 3E, representative trace), which further indicates a specific response to metabolizable glucose and not to osmotic shock or other stress from the added sugars.

### Ca^2+^ transients are not well‐coupled between *β*‐cells in adult Zebrafish islets

**Figure 4:**
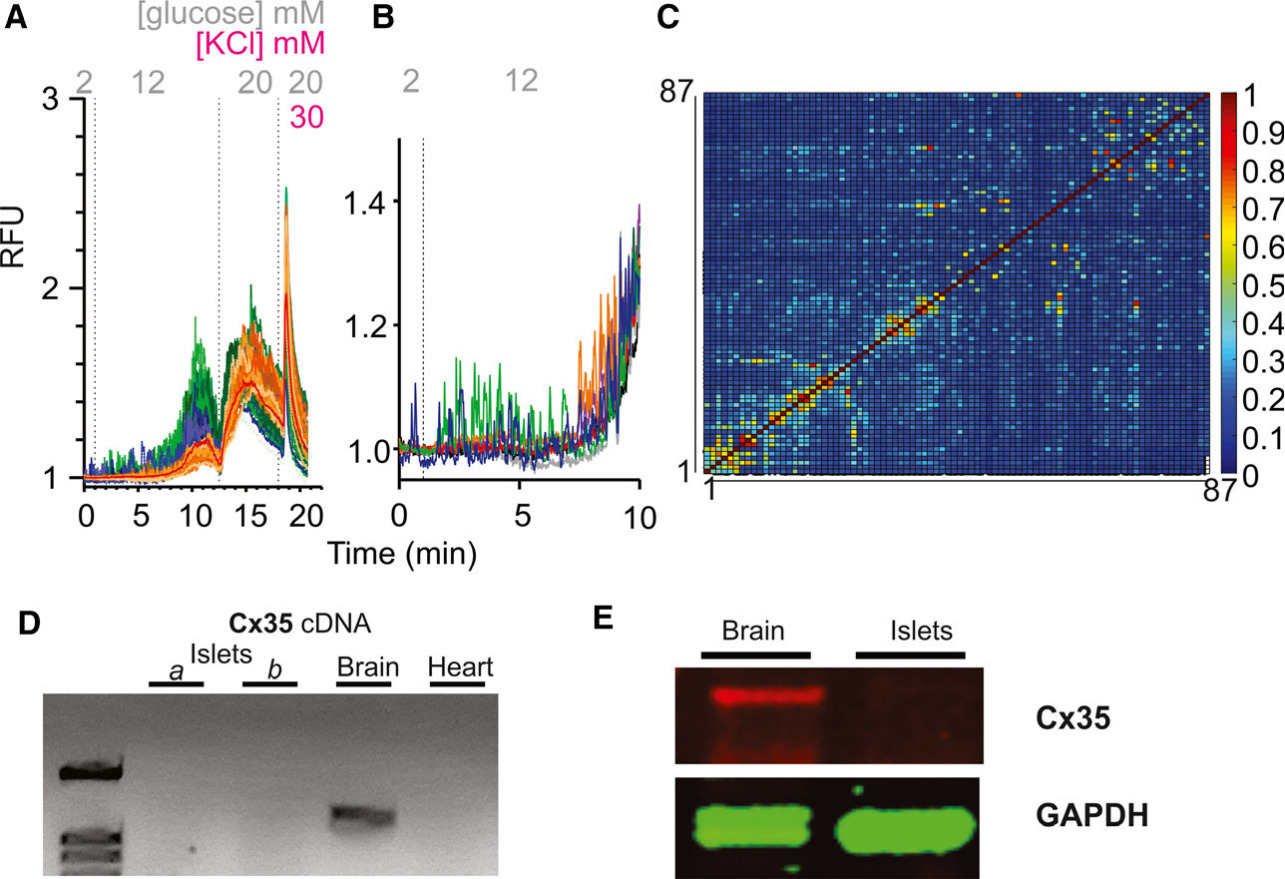
Ca^2+^ transients are not well‐coupled between *β*‐cells in Zebrafish islets. (A) Fluorescence response to switch from 2, to 12, to 20 mmol/L glucose and then KCl. Individual ROI traces are shown for 87 tracked cells from a representative islet, plus averaged trace from the whole islet (red). Some cells (blue) were active at basal (2 mmol/L) glucose. Others activated at 12 mmol/L glucose (green) or only at 20 mmol/L glucose (orange). (B) Representative individual traces from the early 12 mmol/L transition from islet in (A). (C) Cross‐correlation matrix (determined by PeakCaller) of all 87 tracked cells in (A). (D) Representative PCR of Cx35b cDNAs from islets, brains, and hearts of zebrafish. Sets *a* and *b* are different pools of islets (biological replicates). (E) Western blot analysis of zebrafish Cx35 protein. Cx35 is detected in zebrafish brain but not in zebrafish islets.

In mammals, individual *β*‐cells vary in their expression of metabolite transporters, metabolic enzymes, and ion channels involved in the insulin secretion response, and thus exhibit variable sensitivities to glucose (Benninger et al. 2014; Silva et al. 2014). However, in intact islets, gap‐junction coupling between *β*‐cells ensures synchronous electrical and calcium signals, and hence secretory responses, across the islet (Farnsworth and Benninger 2014). In contrast to mammals, uncorrelated gCAMP6s fluorescence oscillations were observed in individual *β*‐cells within the zebrafish islet. In high‐resolution confocal images (Fig. 4A), individual cells clearly become active at very different glucose levels. Even cells that are physically close together lack synchronicity in their calcium spikes and glucose sensitivity (Fig. 4B), evidenced by very weak correlation coefficients between signals from individual cells (Fig. 4C). This is in stark contrast to the strong cell‐cell correlation in isolated mouse islets under comparable conditions (Kenty and Melton 2015; Johnston Natalie et al. 2016).

Connexin 36 is the primary gap junction protein in mammalian islets (Farnsworth and Benninger 2014). Connexin 35b is the major ortholog of mouse connexin 36 in fish (Jabeen and Thirumalai 2013; Carlisle and Ribera 2014; Watanabe 2017). It has been well‐characterized in zebrafish brain (Jabeen and Thirumalai 2013; Carlisle and Ribera 2014), and both cDNA (Fig. 4D) and protein (Fig. 4E) were readily detected in brain, but not in zebrafish islets or heart.

### Islets expressing K_ATP_‐GOF are inexcitable, resulting in profound diabetes

**Figure 5:**
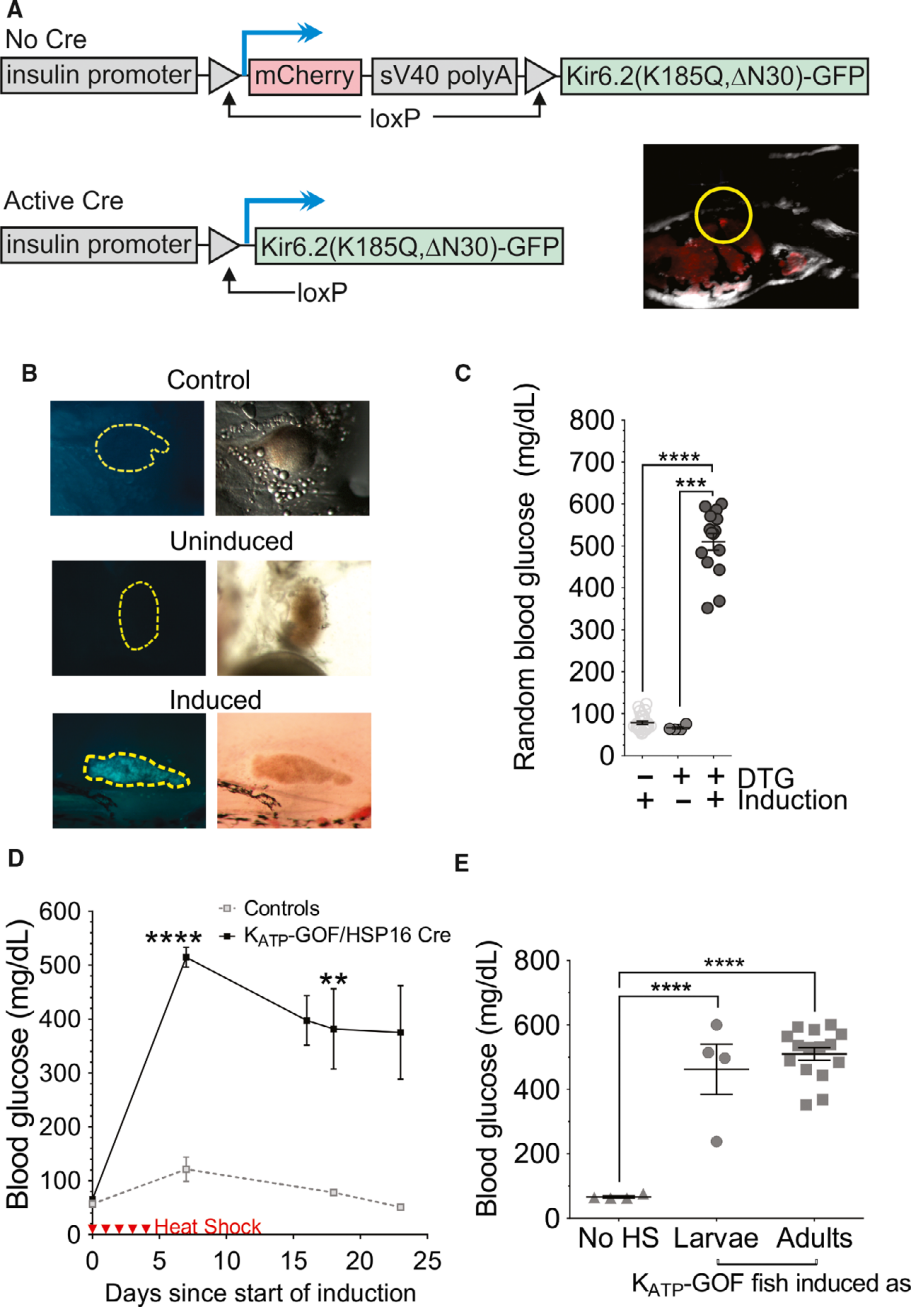
Islet K_ATP_‐GOF results in profound diabetes. (A) Transgenic strategy for conditional K_ATP_‐GOF expression in zebrafish islet. mCherry, expressed under insulin promoter control (upper panel), is excised and K_ATP_‐GOF construct is expressed, after (lower left panel) Cre recombination. A F2 larva is shown in the right lower panel, with the islet highlighted in the yellow circle. (B) GFP (left column) and bright‐field (right column) images of dissected islets from adult control, uninduced K_ATP_‐GOF and induced K_ATP_‐GOF fish (images taken at 12 × ). (C) Random blood glucose levels in controls (*N* = 25), uninduced (*N* = 15), and induced (5 days heat‐shock, *N* = 24) K_ATP_‐GOF zebrafish. In control and induced fish, blood glucose were measured 2 days after the last heat shock. (D) Time course of change in blood glucose following K_ATP_‐GOF induction. (E) Glucose levels in adult (10 week old) K_ATP_‐GOF fish are similarly elevated, whether induced as larvae (*N* = 4), or as adults (*N* = 15).

In mammals, excitability is dramatically suppressed by gain‐of‐function mutations in K_ATP_ channels, resulting in profound neonatal diabetes (Koster et al. 2000; Gloyn et al. 2004). To examine susceptibility of zebrafish glycemia to *β*‐cell membrane excitability, we generated additional transgenic fish (Tg(‐1.0ins:LoxP_mCherry_polyA_LoxP,Kir6.2(K185Q,∆N30)‐GFP)^stl443^, K_ATP_‐fish) which conditionally express the same GFP‐tagged gain‐of‐function Kir6.2 subunit that has been extensively used to demonstrate and study neonatal diabetes in mice (Koster et al. 2000) and previously shown to increase glucose levels in zebrafish larvae (Li et al. 2014). We crossed these K_ATP_‐fish to zebrafish expressing HSP‐16 inducible Cre‐recombinase, to generate K_ATP_‐GOF animals which express the K_ATP_‐GOF transgene only in *β*‐cells, following heat shock induction (schematic in Fig. 5A). Robust transgene expression is evident in double transgenic *β*‐cells after 5‐days heat‐shock (by visualizing tagged GFP, Fig. 5B).

Following 5 days of heat shock induction, blood glucose was unaltered in non‐GOF control or single transgenic fish/islets (Fig. 5C), but K_ATP_‐GOF fish rapidly developed severe hyperglycemia (>600 mg/dL), and this was then maintained >400 mg/dL for weeks (Fig. 5D). When the transgene was activated at the larval stage, glucose levels in adult fish were similar to those resulting from adult induction (Fig. 5E)

**Figure 6:**
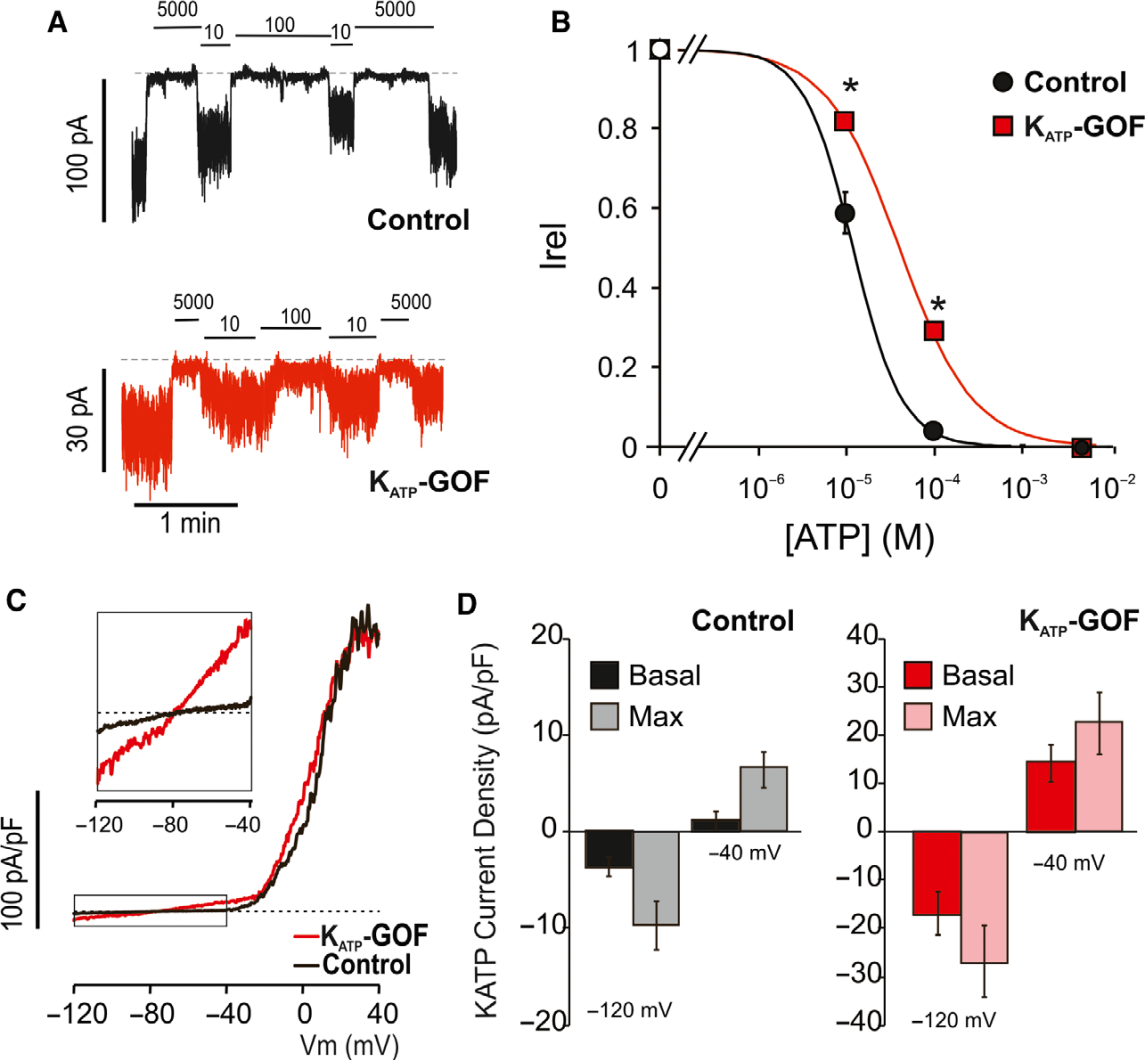
Islet K_ATP_‐GOF expression results in basal K_ATP_ and ATP‐insensitive channels. (A) Representative excised inside‐out patch‐clamp recordings (at −50 mV) from *β*‐cells isolated from control (black) or K_ATP_‐GOF (red) islets, in the presence of ATP at concentrations (in micromolar) as indicated. (B) Steady‐state dependence of membrane current on [ATP] (relative to current in zero ATP (I_rel_)) for control and K_ATP_‐GOF channels. Data points represent the mean ± SEM. (*n* = 4 patches in each case). The fitted lines correspond to least squares fits of a Hill equation (see Methods). (*) *P* < 0.01 compared to wild‐type K_ATP_ (controls) by unpaired Student's *t* test. (C) In whole‐cell mode basal conditions, voltage‐clamp ramps from −120 to −40 mV (over 1 sec) activates similar Kv currents above −20 mV in both K_ATP_‐GOF and control cell. However, basal K_ATP_ channel activation is evident in K_ATP_‐GOF cells as additional ~linear current reversing at −80 mV (boxed current is amplified in insert). (D) Averaged basal currents at −120 and −40 mV from experiments as in C (*n* = 5 control cells, *n* = 7 K_ATP_‐GOF cells).

**Figure 7:**
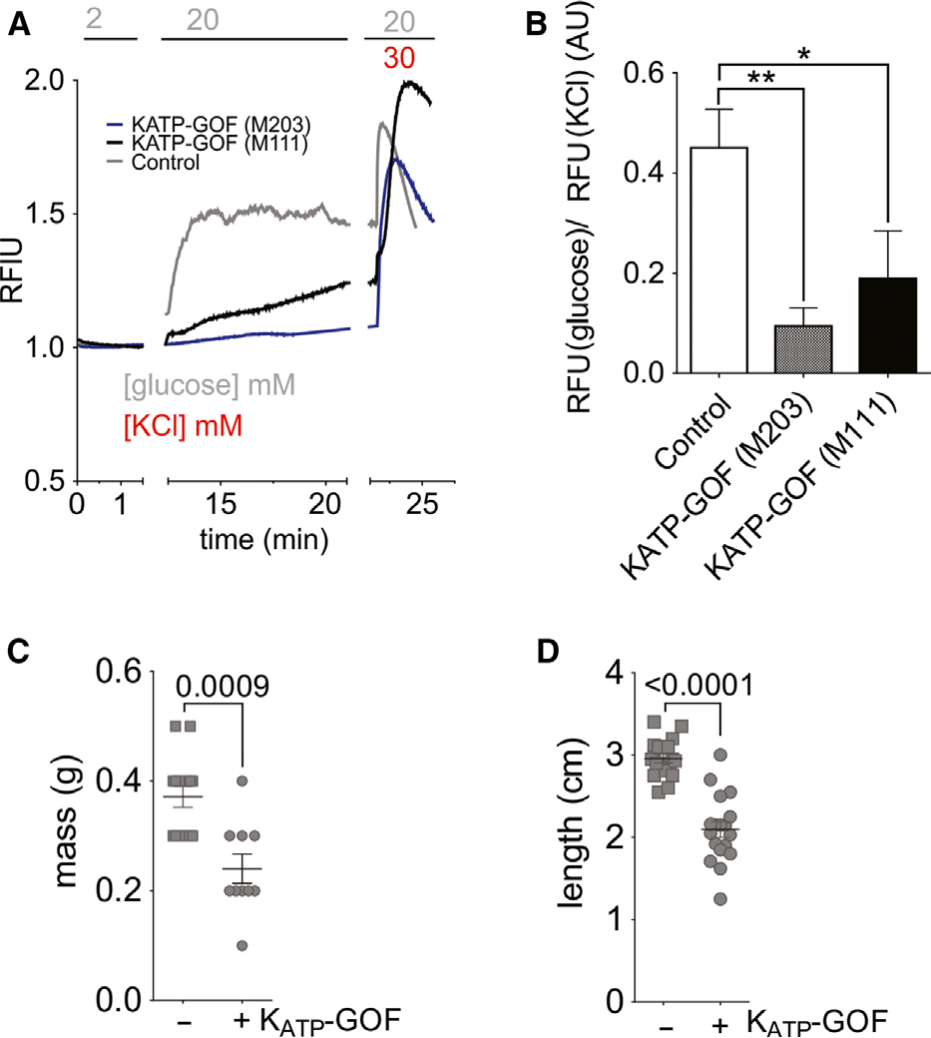
K_ATP_‐GOF inhibits glucose‐dependent Ca and causes secondary diabetic complications. (A) Representative recording of intracellular calcium response to switch from 2 to 20 mmol/L glucose in control (gCAMP6s only) and K_ATP_‐GOF/gCAMP6s islets from two different founder lineages (M203, M111). (B) Average calcium response to 20 mmol/L glucose from control (*n* = 8) and two different founder lineages (M203, *n* = 8 and M111, *n* = 7) of K_ATP_‐GOF/gCAMP6s fish, normalized to absolute calcium response to depolarization in KCl. (C) Body mass (*N* = 10–14) and (D) body length (*N* = 18) in K_ATP_‐GOF (+) and control (−) fish that were induced as larvae. B, C, and D are analyzed by 1‐way ANOVA followed by Tukey's post‐tests. (*) *P* < 0.05, (**) *P* < 0.01.

Excised inside‐out patch‐clamp experiments (Fig. 6A) confirm that, in fluorescent cells, the K_ATP_‐GOF transgene was incorporated into *β*‐cell K_ATP_ channels, resulting in the expected loss of ATP‐sensitivity (Fig. 6B). Even though overall channel density was if anything reduced (excised patch current in zero ATP was 51.8 ± 7.4 pA in control, *n* = 4, c.f. 9.6 ± 2.4 pA in K_ATP_‐GOF, *n* = 4), whole‐cell voltage‐clamp currents (Fig. 6C) show that, under basal conditions following break‐in, voltage‐gated K currents were unaffected in K_ATP_‐GOF islets, but K_ATP_ currents were already basally activated (Fig. 6C and D). To examine the consequences for glucose‐dependent Ca‐handling, we crossed K_ATP_‐GOF fish to cGCAMP6s fish. Isolated islets from heat‐shock induced K_ATP_‐GOF fish show essentially no glucose‐induced calcium responses, even at high (20 mmol/L) glucose levels, although they still respond appropriately to direct depolarization by 30 mmol/L KCl (Fig. 7A and B). These results are consistent with K_ATP_‐GOF inhibiting electrical activity at high [glucose] by hyperpolarizing cells, an effect that is overcome by direct KCl‐induced depolarization.

K_ATP_‐GOF mice with untreated diabetes develop significant secondary consequences, including growth limitation (Girard et al. 2009; Remedi et al. 2009). Larvae‐induced K_ATP_‐GOF fish also showed dramatically reduced body length and weight at 10 weeks of age, when compared to Cre‐negative littermate controls from the same clutches (Fig. 7C and D). These data indicate that not only does *β*‐cell K_ATP_‐GOF induce similar inexcitability‐dependent hyperglycemia in zebrafish as in mammals, but also similar secondary diabetic consequences.

## Discussion

### Conservation of islet function between mammals and fish

In mammals, excitability‐dependence of intracellular [Ca^2+^] is well‐established and is shown to be critical for regulation of insulin secretion. By contrast, islet excitability in lower vertebrates remains essentially unaddressed. We previously reported the expression and function of K_ATP_ channels in zebrafish islet *β*‐cells and showed that pharmacological K_ATP_ channel activators worsened glucose tolerance in adult fish (Emfinger et al. 2017). Larval activation of K_ATP_‐GOF transgene under tetracycline and tebufenozide‐driven promoter control in *β*‐cells, or treatment of normal larvae with diazoxide, raises glucose levels and inhibits overnutrition‐induced *β*‐cell expansion (Li et al. 2014), and intracellular [Ca^2+^] is glucose‐dependent in larval zebrafish islets (Lorincz et al. 2018). In the present study we have now characterized glucose‐dependence of intracellular calcium in adult zebrafish islets, and show that the glucose dependence of calcium oscillations is similar, although not identical, to mammalian islets. We further show that islet calcium response to glucose can be blocked by *β*‐cell specific induction of GOF mutations in K_ATP_, resulting in profound diabetes. This indicates that key components of excitability‐dependent insulin secretion are well conserved between mammals and fish, although unfortunately, we do not have a suitable assay for secreted insulin or C‐peptide in zebrafish.

However, we observe several potentially important differences. First, amplification of glucose signals by amino acids, which is observed in mammalian islets (Henquin et al. 2006; Liu et al. 2008) and embryonic zebrafish islets (Lorincz et al. 2018), was not seen in adult fish islets (Fig. 3D). Given that zebrafish may be more dependent on protein than carbohydrate in the diet, this is teleologically surprising, but may reflect a difference in cellular expression of essential carriers or enzymes. Second, isolated zebrafish islets are not well‐coupled electrically (Fig. 4). Tight coupling of *β*‐cells to one another, evidenced by tight cell‐cell coupling of Ca^2+^ transients, is critical for normal mammalian glucose tolerance (Klee et al. 2008; Farnsworth and Benninger 2014): in mice lacking connexin 36, which forms the primary gap junctions in islet *β*‐cells, basal insulin is elevated, and glucose responses of the overall islet are slowed, worsening glucose tolerance in otherwise healthy animals (Head et al. 2012). Because of the lack of cell‐cell coupling in the adult fish islets, glucose‐sensitivity of Ca^2+^ is quite variable between cells within the intact islet (Fig. 4). Variable glucose‐sensitivities of *β*‐cells ex vivo have also been observed in larval and early juvenile zebrafish (Singh et al. 2017; Lorincz et al. 2018); ex vivo recordings of these younger zebrafish islets clearly show cells activating independently, at thresholds between 5 and 20 mmol/L glucose. Absence of cell‐cell electrical coupling in zebrafish *β*‐cells may explain, in part, the relatively lower glucose tolerance of zebrafish compared to mammals (Eames et al. 2010; Emfinger et al. 2017), with higher peak blood glucose and slower return to baseline glucose after a glucose injection.

### K_ATP_‐GOF zebrafish recapitulate major features of mammalian neonatal diabetes

Transgenic mice expressing K_ATP_‐GOF at birth, as well as human K_ATP_‐dependent human neonatal diabetic patients, exhibit reduced growth rates (Koster et al. 2000; Polak and Cave 2007; Girard et al. 2009; Remedi et al. 2009). Mammalian and Danio K_ATP_ genes are very similar (>90% identity at the amino acid level) (Emfinger et al. 2017). K_ATP_‐GOF zebrafish, expressing the identical transgene that we originally used in mice (Koster et al. 2000), results in basal activity of K_ATP_ channels in *β*‐cells and in the fish becoming profoundly hyperglycemic, and also showing growth limitation when induced as larvae. Other secondary consequences of hyperglycemia can occur directly in the islet, with loss of *β*‐cell identity and insulin content (Brereton et al. 2014; Wang et al. 2014). Whether this occurs in zebrafish islets exposed to high glucose for long periods is currently unknown but future lineage tracing studies using models such as ours may now be used to clarify these questions.

Zebrafish are transparent as larvae, reproduce frequently and in large clutches, have a fully sequenced genome, and can be efficiently genetically mutated, making them well‐suited to large‐scale drug or genetic screens. We have shown that zebrafish *β*‐cells exhibit many similarities to mammals in glucose responsivity, including the profound hyperglycemia that results from electrical glucose‐unresponsivity. We thus provide a zebrafish model of *β*‐cell inexcitability‐dependent diabetes that may be useful for drug and genetic modifier screens.

## Ethics Statement

All procedures were approved by the Washington University Institutional Animal Care and Use Committee.

## Conflict of Interest

None.
